# Situs Inversus Totalis in Laparoscopic Sleeve Gastrectomy: A Case Report

**DOI:** 10.7759/cureus.46539

**Published:** 2023-10-05

**Authors:** Milton Alberto Muñoz-Leija, Guillermo Álvarez-Valdés, Gabriel Rosales-Pérez

**Affiliations:** 1 Endobariatric Division, Endohospital, Piedras Negras, MEX

**Keywords:** laparoscopic sleeve gastrectomy, #laparoscopic sleeve gastrectomy, obesity, bariatric surgery', situs inversus totalis (sit), gastric sleeve surgery

## Abstract

Obesity is a pathology that is increasing in incidence globally and threatens public health. Currently, one of its most effective treatments is bariatric surgery, which has shown the best long-term results. One of the most frequently performed surgical procedures in this area is laparoscopic sleeve gastrectomy (LSG) or vertical sleeve. It is a restrictive technique that has had positive results in weight loss. Situs inversus totalis (SIT) is a strange condition with a low incidence in which thoracic and abdominal organs are on the opposite side of the already-known anatomic site, seen on a sagittal plane. The high demand for laparoscopic bariatric surgeries predisposes surgeons to find rare congenital anomalies in patients. Low prevalence and the anatomic mirror image condition may be challenging even for expert surgeons. Medical teams need to have knowledge of these cases and adjust the technique and procedure to complete the surgery without complications. We present a SIT case successfully treated with LSG in a male patient in Mexico.

## Introduction

Obesity is one of the most important epidemics of the current century worldwide. It is associated with other highly prevalent comorbidities such as hypertension, diabetes mellitus, non-alcoholic fatty liver disease, and other metabolic syndromes, reducing patients' quality of life and life expectancy [1]. With each passing year, bariatric surgeries are becoming more common, especially due to the great benefits related to weight loss [2]. One of the most popular bariatric procedures is laparoscopic sleeve gastrectomy (LSG) because of its simplicity and decreased rate of complications. The average surgery duration ranges from 35 to 50 minutes [3].

Situs inversus totalis (SIT) is a rare genetic autosomal recessive congenital anomaly in which intrathoracic and intra-abdominal organs are positioned in a mirror image. It is estimated to be present in 0.01% of the population. Most of the patients affected have normal health and life expectancy. However, this can be associated with respiratory, cardiovascular, or digestive anomalies [4].

The increase in obesity worldwide and patients' preference for bariatric surgery due to its excellent results [5], as well as the increase in medical tourism, predispose surgeons to find patients with low-incidence congenital anomalies, such as SIT. This article reports a rare case of SIT in a male patient treated successfully by LSG in Mexico. This case has been reported in line with the Surgical CAse REport (SCARE) criteria [6]. 

## Case presentation

**Figure 1:**
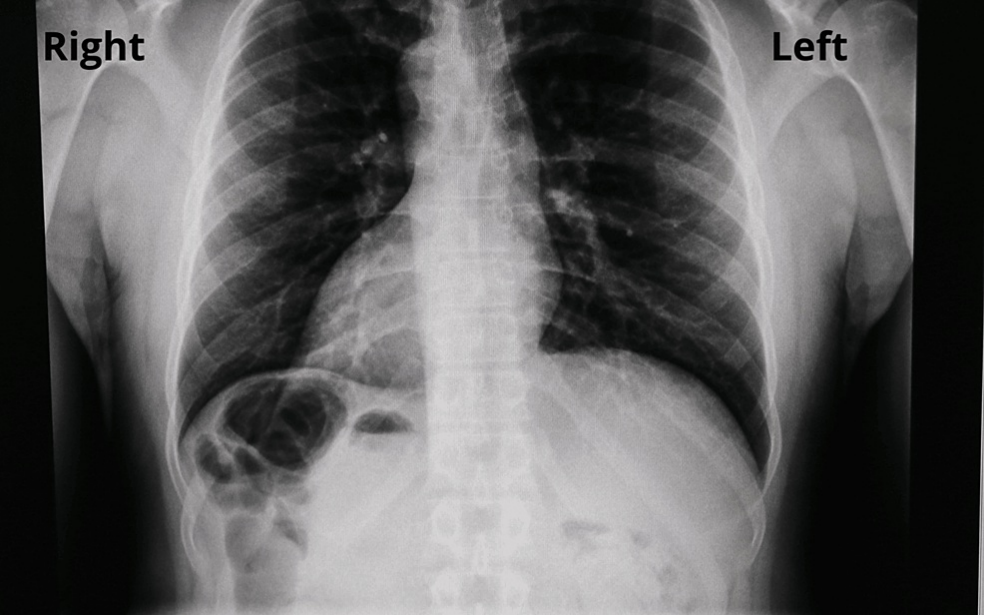
Chest X-ray. Organs are found in an inverted position.

A 50-year-old male patient with a body mass index (BMI) of 36.3, medical history of SIT, and left inguinal hernia repair presented to our hospital for a gastric sleeve (GS) surgery eligibility evaluation. The patient has been undergoing a strict exercise and diet regimen for a year, including the use of orlistat and sibutramine, both prescribed by a physician from his country of origin (United States). After nutritional guidance for three months by our physicians, the laboratory workup revealed the following results: white blood cell counts 7.36 x 10^9^/L, hemoglobin 14.5 g/dL, international normalized ratio (INR) 1.02, aspartate aminotransferase (AST) 34.5 U/L, and alanine transaminase (ALT) 39 U/L. Chest X-ray showed dextrocardia and a stomach bubble under the right hemidiaphragm (Figure 1).

**Figure 2:**
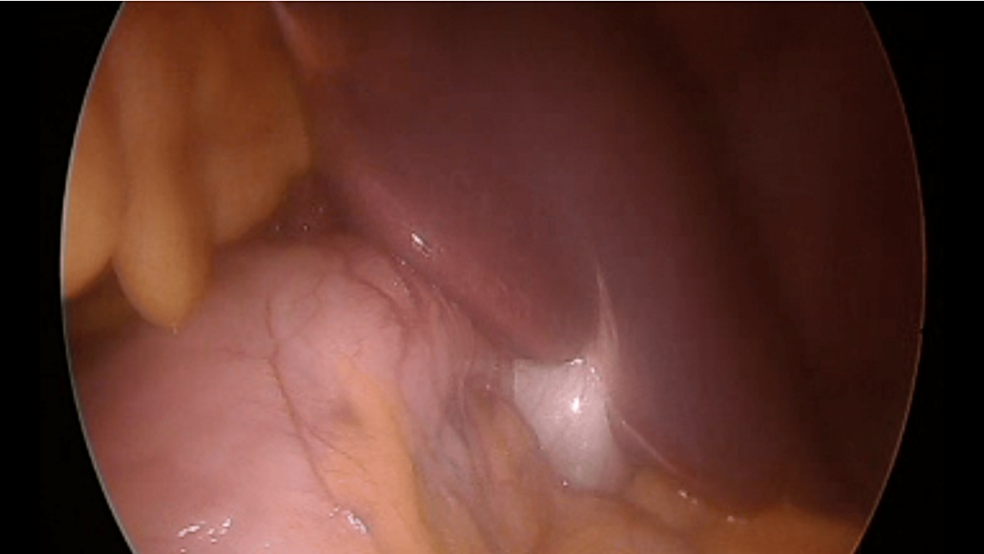
Laparoscopic view of the intra-abdominal organs with the liver and gallbladder on the left upper quadrant.

LGS was performed under general anesthesia. The patient was positioned on decubitus with the French position. The surgeon stood on the right side of the patient, and the assistant was on the left side. Using a Veress needle, insufflation was performed at Palmer's point (3 cm below the left costal margin in the midclavicular line), achieving a rise in pneumoperitoneum pressure to 15 mmHg. A 10 mm trocar was positioned on the supraumbilical midline, the camera was introduced through it, and then diagnostic laparoscopic surgery was performed (Figure 2).

**Figure 3:**
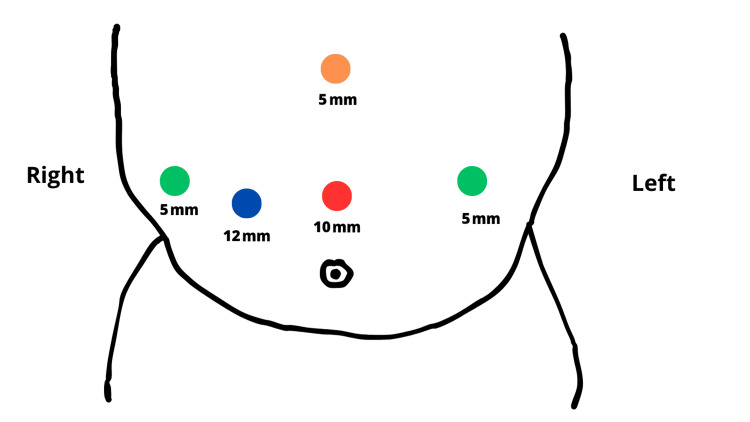
Port placement diagram demonstrating the placement of a 10 mm trocar for camera in the supraumbilical midline (red dot), a 12 mm trocar for staples at the supraumbilical right clavicular midline (blue dot), a 5 mm trocar for liver retractor at the subxiphoid midline (orange dot), a 5 mm trocar on the right anterior axillary line (green dot), and a 5 mm trocar on the left anterior axillary line (green dot).

Under direct vision, a 5 mm trocar was placed on the right anterior axillary line and another 5 mm trocar on the left side. A subxiphoid 5 mm trocar was placed in the midline for the liver retractor, and a 12 mm supraumbilical trocar was placed along the right midclavicular line for staples (Figure 3).

**Figure 4:**
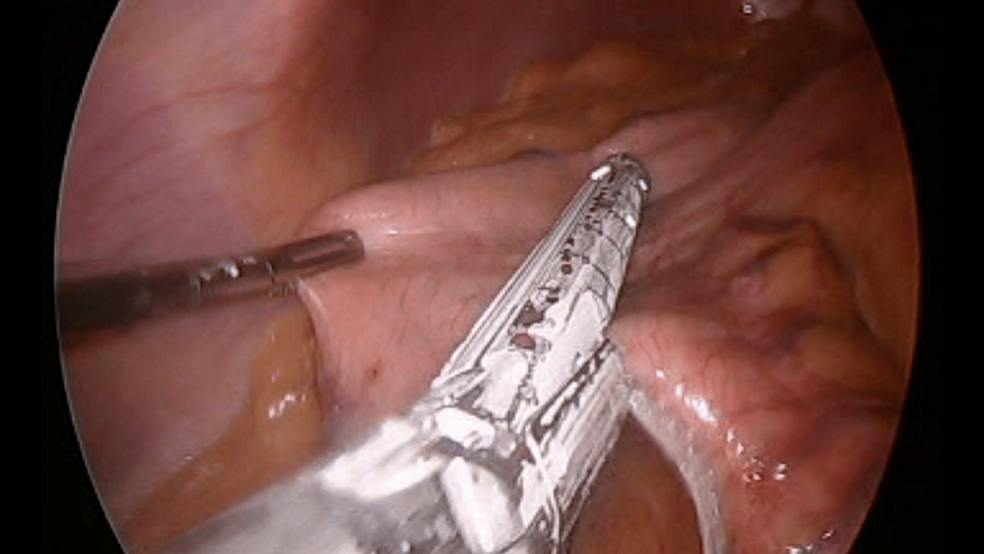
Stomach stapling.

The greater curvature of the stomach was released approximately 5 cm from the pylorus. Using a bipolar energy device, it was released up to the gastroesophageal junction, taking care of the hemostasis of the short vessels. The omentum was detached, and the fundus was mobilized. A 32-Fr gastric calibration tube was passed toward the first portion of the duodenum. For the new stomach, a surgical stapler (Medtronic, Dublin, Ireland) was used under direct vision (Figure 4). 

**Figure 5:**
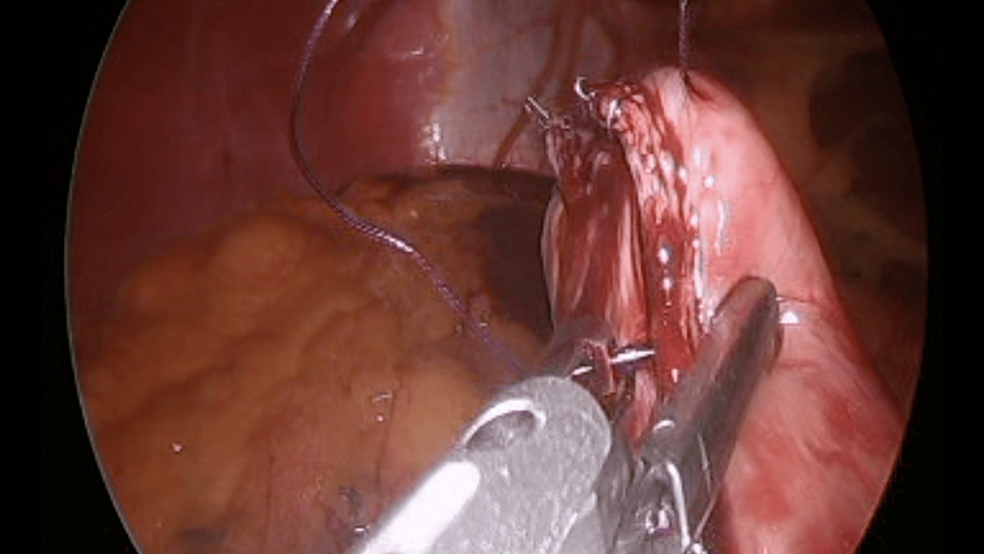
Staple line reinforcement.

The sleeve was completed, and the gastric tube was removed. The staple line was then reinforced with Endostitch™ (Covidien, Dublin, Ireland) using an interrupted absorbable suture (Figure 5).

The supraumbilical incision was closed with M-CLOSE Kit™ (New Wave Endo, Coconut Creek, FL, United States) with 10 mL of ropivacaine 7.5 mg/mL and an absorbable suture. The skin was closed with a monofilament suture. The procedure was completed in 30 minutes.

The first 24-hour postoperative period was uneventful, and a strict ice-only diet was started without complication. The patient was discharged on postoperative day 2 with a liquid diet for one week. With good evolution and liquid tolerance found during the one-week follow-up visit, the diet was changed to a soft diet and nutrition follow-up. ﻿Six months after surgery, the patient has lost approximately 40% of his excess weight. 

## Discussion

SIT is a rare congenital anomaly with a very low incidence, occurring in less than 0.01% of the population. It is described as the transposition of organs to the opposite side of the body. Probable etiologies of this disease have been reported; however, its genetic origin remains unknown. The literature suggests an X-chromosome defect with an autosomal recessive pattern [7,8].

The first reported data available about this condition are attributed to Aristotle; however, he reported it on animals. The first report of this condition in humans was made in the 16th century [9-11]. However, it was first defined in the 18th century by Matthew Baillie, a British physician and pathologist, with his students in England (Hunterian School of Medicine), as a mirror image of normal organ placement [7,8].

In bariatric surgery, the first case was reported in 1998, and since then, only 40 cases of LGS in SIT patients have been published in the literature [12,13]. To the best of our knowledge, only two female patients have been reported in our country (northwest Mexico) [14,15]. This is the first case of a male patient who underwent GS surgery with SIT in Mexico.

The concept of "centers of excellence" was created to establish quality indicators that must be met in large bariatric centers using recording and reporting data on the surgical procedures performed [16]. Our hospital, located in the northeast of the country near the border with the USA, has a center of excellence and has performed approximately more than 20,400 bariatric procedures on patients from neighboring countries with excellent results since 2018. The continuous evolution of bariatric procedures with the incorporation of new technological advances allows for favorable results and avoids possible complications in patients [17]. Endostitch™ (Covidien, Dublin, Ireland) is one of the recently included tools for bariatric surgery in our country. However, its use has been described in the laparoscopic surgery literature [18]. M-CLOSE Kit™ (New Wave Endo, Coconut Creek, FL, United States) is another technological device recently introduced in our country that has presented favorable results in our patients. Prospective studies to evaluate these two devices are necessary to establish their effectiveness. 

Bariatric surgery is one of the best treatments for obesity, and its effectiveness in diseases of metabolic origin, such as diabetes, has already been well described in the literature [19]. A greater number of patients are undergoing a bariatric surgical procedure, especially with the increasing amount of medical tourism around the world. This predisposes to a rise in the probability of finding patients with low-incidence diseases. Therefore, the bariatric surgeon must be aware of this type of case to prepare a safe surgical procedure, choose the best technique, avoid complications, and ensure adequate postoperative treatment.

## Conclusions

Although most of the patients with SIT do not present symptoms, they must be identified and adequately evaluated during the preoperative period. The bariatric surgeon must be familiar with this type of case, especially in centers of excellence. Promising success rates have been reported in the literature when performing LGS in patients with SIT, suggesting that this condition is not a contraindication for surgery. The surgeon's knowledge and experience are key factors in reducing the risk of complications.
